# Protective effects of the fruit extract of raspberry (*Rubus fruticosus* L.) on pituitary-gonadal axis and testicular histopathology in streptozotocin induced diabetic male rats

**Published:** 2021

**Authors:** Nooshin Amini, Abdolhossein Shiravi, Naser Mirazi, Vida Hojati, Roghayeh Abbasalipourkabir

**Affiliations:** 1 *Department of Biology, Damghan Branch, Islamic Azad University, Damghan, Iran *; 2 *Department of Biology, Faculty of Basic Sciences, Bu- Ali Sina University, Hamedan, Iran*; 3 *Department of Clinical Biochemistry, School of Medicine, Hamedan University of Medical Sciences, Hamedan, Iran*

**Keywords:** Diabetes, Male reproductive system, Nitric oxide, Malondialdehyde, Superoxide dismutase, Catalase

## Abstract

**Objective:**

Protective effects of raspberry (*Rubus fruticosus* L.) fruit extract on pituitary-gonadal axis and testicular tissue in diabetic male rats, were investigated.

**Materials and Methods:**

Sixty male rats were divided into control, sham (saline treated), streptozotocin (STZ)-diabetic, and STZ-diabetic animals treated with 50, 100 and 200 mg/kg/day of raspberry extract. After 4 weeks, blood samples were obtained and left testes were removed and prepared for histopathological studies. Serum levels of Luteinizing hormone (LH), Follicle stimulating hormone (FSH), testosterone, Nitric oxide (NO), and malondialdehyde (MDA), as well as superoxide dismutase (SOD) and catalase (CAT) activity level were assayed. Sperm number and motility in the epididymis samples were measured. Data were analyzed using ANOVA (one-way analysis of variance).

**Results:**

Serum levels of LH, FSH and MDA significantly increased in diabetic rats, however, treatment with the extract significantly reversed the alterations. Serum levels of testosterone and NO, activity of SOD and CAT, and sperm number and motility significantly decreased and severe destruction of testicular histology was observed in diabetic animals while treatment with the extract significantly reversed the pathologic alterations observed in diabetic rats. According to the results, 100 and 200 mg/kg of the extract were able to effectively reverse the diabetes complications.

**Conclusion:**

Our findings demonstrated that the fruit extract of raspberry has protective effects on male reproductive system in diabetic rats partially due to its improving effects on NO system, and SOD and CAT activity.

## Introduction

Defective discharge or deficiency in insulin function in diabetes leads to chronic hyperglycemia which affects and degrades the function of various organs, including the kidneys, nerves, heart, blood vessels and testicles (Haas et al., 2010 ▶). Studies on diabetic animals showed that hyperglycemia leads to reduced reproductive performance (La Vignera et al., 2012 ▶). Reduced sperm count and motility, and testosterone level, as well as sexual dysfunction and various degrees of testicular lesions were reported in diabetic animal models (Zha et al., 2018 ▶). It was found that diabetes tends to reduce spermatogenesis by inducing changes, including oxidative stress and free radical production (Asadi et al., 2017 ▶) and acting on nitric oxide (NO) system in the seminiferous tubules and reducing the number of spermatogonia cells (Moodley et al., 2015 ▶). To detect oxidative stress in tissues, malondialdehyde (MDA), a carbonyl group produced during lipid peroxidation, is widely studied in experimental studies (Soydinç et al., 2007 ▶). Superoxide dismutase (SOD) is also one of the key enzymes for the antioxidant defense of the body against free radicals-induced damage. This enzyme accelerates the conversion of two radicals of anion superoxide to hydrogen peroxide and molecular oxygen, and thus scavenges the harmful radicals (Gopal and Elumalai, 2017 ▶). Catalase (CAT) plays an important role in the removal of hydrogen peroxide. This enzyme effectively converts hydrogen peroxide into water and an oxygen molecule (Nimse and Pal, 2015 ▶). Nitric oxide (NO), as a mediator, plays an important role in many functions of the nervous system (Taherian et al., 2010 ▶). Various studies revealed the role played by NO in normal functioning of the male and female reproductive system (Hassanpour, 2007 ▶).

Raspberry (*Rubus fruticosus* L.) has a broad spectrum of protective functions against diverse degenerative diseases. *R. fruticosus* L. contains vitamins such as A, C, and E, folic acid, mineral chloride, Zn, Cu, Al, Mn, Co, and Fe as well as various types of sterols (Zia-Ul-Haq et al., 2014 ▶) which contribute to protective function of the plant against a wide variety of disorders. Different types of carotenoids, marinova, β-cryptoxanthin, lycopene, zeaxanthin, β-carotene and α-carotene were also extracted from the fruit of *R. fruticosus*. Raspberry is a rich source of natural antioxidants because it contains large quantities of phenols, flavanols and anthocyanins, which act as free radical inhibitors. The most important effects of raspberry are antimicrobial, antioxidant, anti-inflammatory and anti-cancer functions (Zia-Ul-Haq et al., 2014 ▶).

A vast majority of research concerning raspberry is focused on its chemoprevention and anticancer effects, and few research were carried out to detect the protective effects of raspberry extract, particularly fruit extract, on the male reproductive system in diabetic subjects.

Due to the fact that the fruit of the raspberry plant has antioxidant properties and can prevent the side effects of oxidative stress caused by diabetes (Zia-Ul-Haq et al., 2014 ▶), the main aim of this study was to determine the protective effects of the fruit extract of raspberry (*Rubus fruticosus* L.) on pituitary-gonadal axis, and serum levels of NO, and MDA, and SOD and CAT activity in streptozotocin (STZ)-diabetic male rats.

## Materials and Methods

The study protocol was approved by the Ethics Committee of Hamedan University of Medical Sciences (IR.UMSHA.REC.1399.414).

**Experimental design**

To investigate the anti-diabetic effects of fresh fruits of raspberry extract, we carried out an *in vivo* study to show whether the extract may reverse diabetes effects on pituitary-gonadal axis and testicular histopathology in STZ-induced diabetic male Wistar rats. Serum levels of LH, FSH, testosterone, NO, and malondialdehyde, as well as superoxide dismutase and catalase activity were assayed and sperm number and motility in the epididymis samples were measured in diabetic animals treated with the extract and compared with control (untreated) rats.

**Extraction**

Fresh fruits of raspberry (*Rubus fruticosus* L.) were obtained from Rudbar County of Gilan Province and authenticated scientifically by the Department of Biology, Malayer University, Malayer, Iran. Voucher specimens of the plant are deposited in the Herbarium of University of Malayer (Malayer, Iran, No. 1036). After desiccation, the fruits under shade, were crushed and powdered using an electric grinder. Then, 500 g of fruit powder was mixed with 80% ethyl alcohol and 20% distilled water and kept in a bain-marie at 50 to 55°C for 72 hr. After purifying the supernatant using filter paper, the solution was extracted by a rotary evaporator machine under a vacuum evaporation condition. Then, under a fume hood, the extract was concentrated for 24 hr, and finally, the desired doses of extract (99%) were prepared by dissolving twice in distilled water (Gomar et al., 2015 ▶).

**Determination of total phenol and flavonoid contents**

To measure total phenolic content of the extract, 0.5 ml of the extract was mixed with 5 ml of Folin Ciocalteu reagent and 4 ml of carbonate sodium solution. After 15 min, the total phenols were measured using spectrophotometry method and the calculations suggested by Karamian et al. (Karamian et al., 2013 ▶). 

Aluminum chloride colorimetric method was used to measure the total flavonoid contents in fresh fruit of raspberry extract. Here, 0.5 ml of extract was mixed with 1.5 ml of methanol, 0.1 ml of 10% aluminum chloride, 0.1 ml of 1 M potassium acetate and 2.8 ml of distilled water. After 30 min, the total flavonoid contents were measured using colorimetric method and calculations suggested by Karamian et al. (Karamian et al., 2013 ▶).

**Animals**

Male Wistar rats (200-250 g) were obtained from Pasteur Institute (Tehran, Iran) and kept under standard conditions (20-25°C; 12 hr light: 12 hr darkness). The animals had free access to tap water and food (Noorafshan et al., 2017 ▶). Experimental trials were done alternatively to find common signs of pathology.

**Protocol of study**

According to previous studies (Mohammadi Mehdiabadihassani et al., 2018 ▶), rats were divided into 6 groups of 10 animals in each group: control (intact) group, sham (normal saline-treated) group, STZ (55 mg/kg), and STZ (55 mg/kg) + raspberry fruit extract (50, 100 and 200 mg/kg) treated rats. STZ (Zanosar Pharmacia and Upjohn Co., Kalamazoo, MI, USA) was dissolved in citrate buffer (pH=7.3) and injected intraperitoneally in animals after a 12-hr fasting period. Three days after STZ injection, blood samples were obtained from the lateral tail vein and analyzed for glucose levels (Biomine, Rightesttm GM300, Biomine Corporation, Switzerland). Rats with blood glucose higher than 250 mg/dl, were considered diabetic. Three days after STZ injection, type 1 diabetes (Shiravi and Sayyad Zomorrodi, 2015 ▶) was considered. The extracts were injected intraperitoneally for 4 weeks on a daily basis. All animal experiments were carried out using the standards in accordance with Guidelines for Ethical Conduct in the Care and Use of Animals.

**Preparation of tissue samples**

The left testes were removed completely after the surgical operation. The epididymis of each testis was also carefully removed. The testes were fixed in 10% formalin and finally the tissues were embedded in paraffin. Slices of 5-μm diameter were stained with hematoxylin-eosin and observed under a light microscope according to details suggested by Johari et al. (Johari et al., 2010 ▶).

**Determination of sperm count and motility**

To obtain the total sperm count in samples, semen was sucked from the cauda epididymis. The semen sample was then diluted using normal saline. The sperm count was performed using a Neubauer counting chamber under light microscope (Sookhthezari et al., 2016 ▶). For sperm motility evaluation, diluted semen samples were mixed with 2.9% sodium citrate dehydrate solution and a drop of the fluid was transferred on to a cover glass and finally, the sperm motility was evaluated using a microscopic method.

**Hormone and enzyme assay**

Blood samples for serum preparation were centrifuged at 3500 rpm for 15 min. Serum levels of testosterone and gonadotropins (FSH and LH) were assayed using ELISA method by standard kits (Marburg, Germany). MDA, SOD and CAT activity was measured using ELISA kit from the ZellBio GmbH (Germany), according to the manufacturer’s instructions.

**Data analysis**

The data were analyzed using one-way analysis of variance (ANOVA) followed by Tukey’s *post-hoc* test. Differences among groups were considered significant when p<0.05.

## Results

**Compounds identified in the extract**

Total phenol and flavonoid contents of the *Rubus fruticosus* L. extract are shown in Table 1.

**Table 1:** Total phenol and flavonoid contents of the *Rubus fruticosus L*. extract

Extract	Total phenol (mg/g dw)	Total flavonoid (mg/g dw)
*Rubus fruticosus L*.	42±0.35	31±0.43

**Hormonal evaluation**

Serum levels of LH and FSH were significantly higher while testosterone was significantly lower in the diabetic animals than the control group (p<0.001); However, alterations in the serum levels of the hormones in diabetic rats were reversed by treatment with the extract of raspberry fruit (50, 100 and 200 mg/kg) (p<0.001). The highest dose of the extract (200 mg/kg) had a higher protective effect on serum levels of LH, FSH and testosterone than the lowest dose (50 mg/kg) (p<0.001, p<0.05, p<0.001, respectively) (Table 2).

**Determination of testis size, testis weight, and sperm count and motility**

Testis size and weight significantly decreased in the diabetic animals compared to the control group (p<0.001); however, alterations in testis size and weight were reversed by treatment with the extract of raspberry fruit (Table 3).

Sperm count and motility also significantly decreased in the diabetic animals compared to the control group (p<0.001). Although treatment of diabetic animals with 50 mg/kg of the extract had no significant impact on sperm count and motility compared to the diabetic rats, daily injection of 100 and 200 mg/kg of raspberry fruit extract could significantly reverse the alterations in sperm count and motility in the diabetic animals (p<0.001 and p<0.01, respectively) (Table 3).

**Evaluation of NO level and MDA, CAT and SOD activity**

Serum level of NO and SOD and CAT activity significantly decreased in the diabetic animals compared to the control group, while there was a significant increase in serum level of MDA (p<0.001). Treatment of diabetic rats with 50 and 100 mg/kg of the extract did not significantly alter serum levels of NO compared to the diabetic animals, however, treatment with 200 mg/kg of the extract led to an enhanced NO level comparable to the control animals. Daily injection of 50, 100 and 200 mg/kg in the diabetic animals could significantly reverse the alteration in serum level of MDA in the diabetic animals (p<0.01, p<0.001 and p<0.001, respectively). CAT activity level was reversed in the diabetic rats treated with 100 and 200 mg/kg of raspberry fruit extract (p<0.001), and SOD activity level alteration was also reversed in the diabetic rats treated with 50, 100 and 200 mg/kg of the extract (p<0.001) (Table 4).

We did not observe a significant difference between data obtained from the sham and control groups in our experiments, indicating that the procedure of injection did not have a significant impact on these data.

**Figure 1:**
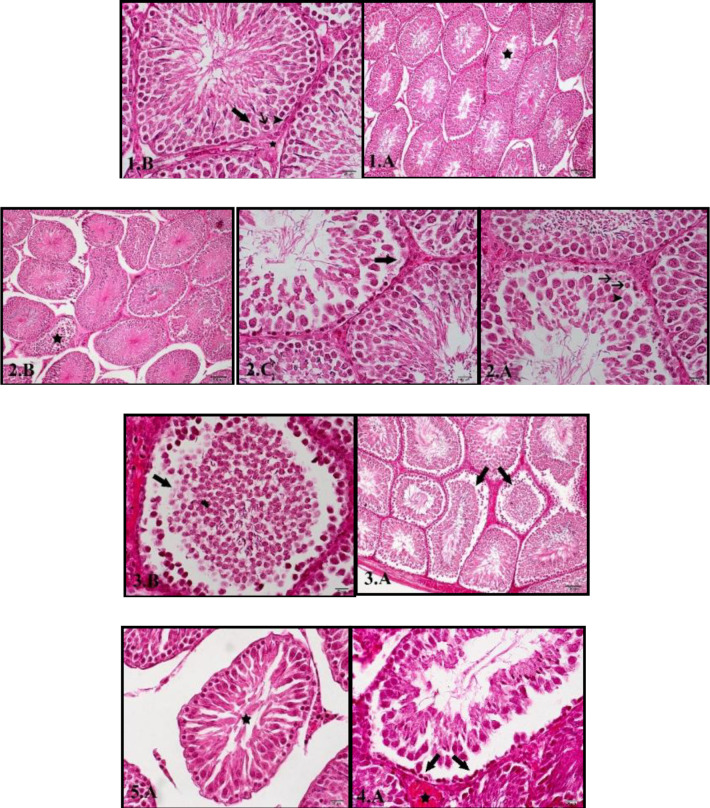
Micrographs of testis from the normal and treated rats. 2: Diabetic animals (DM), 3: DM+Ext (Extract) (50 mg/kg), 4: DM+Ext (100 mg/kg), 5: DM+Ext.(200 mg/kg), 1.A(100×): Seminiferous tubules with normal arrangement of germ cells with an almost empty lumen (asterisk), 1.B(400×): Sertoli cells (large arrow) adjacent to the spermatogonies (small arrow) and peritubular cells (arrowhead). Interstitial tissue with Leydig cells (asterisk), 2.A(400×): Spermatogonial cell apoptosis (arrows) and sloughing of Sertoli cells (arrowhead) next to the peritubular tissue, 2.B(100×): Abnormal shape of seminiferous tubules with detachment of the germ cells and filling up the tubular lumen (asterisk), 2.C(400×): Extensive tubular damage with epithelial atrophy, empty spaces as a result of spermatocytes and spermatids absence (arrow), 3.A(100×) & 3.B(400×): Detachment of the epithelial germ cells (arrows), 4.A(400×): Damaged but regenerating germinal epithelium (arrows) along with interstitial congestion (asterisk), 5.A(400×): Regenerating epithelium and filled lumen (asterisk)

Intertubular edema was observed in histology examination and the epithelial layer of the epididymis was degenerated (Figure 1: 3.A and 3.B). However, treatment of the diabetic animals with 100 mg/kg (Figure 1: 4.A) and 200 mg/kg (Figure 1: 5.A) of the extract significantly reversed the histopathological changes imposed to normal testicular tissue with epithelial reconstruction and increased number of the main cells.

**Table 2:** Serum levels of LH, FSH and testosterone in different groups of male Wistar rats

**Testosterone (ng/ml)**	**FSH (mIU/ml)**	**LH (mIU/ml)**	**Groups **
4.86±0.032	1.52±0.005	1.84±0.010	Control
4.80±0.030	1.57±0.023	1.86±0.023	Sham
2.16±0.013 ***###	2.67±0.039 ***###	2.60±0.008 ***###	DM
3.53±0.019 ***###$$$	1.78±0.019 ***###$$$	2.12±0.010 ***###$$$	DM+Ext. (50 mg/kg)
3.31±0.008 ***###$$$+++	1.75±0.021 ***##$$$	2.17±0.007 ***###$$$+	DM+Ext. (100 mg/kg)
4.79±0.016 $$$+++**†††**	1.67±0.019 **$$$+	1.94±0.014 ***##$$$+++**†††**	DM+Ext. (200 mg/kg)

*indicates a significant difference compared to the control group. # indicates a significant difference compared to the sham group. $ indicates a significant difference compared to the diabetic (DM) group. + indicates a significant difference compared to the DM + extract receiving (EXT; 50 mg/kg) group. **† **indicates a significant difference compared to the DM + Ext (100 mg/kg) group. (** p<0.01 and *** p<0.001); (## p<0.01 and ### p<0.001); ($$$ p<0.001); (+ p<0.05 and +++ p<0.001) and (**†††** p<0.001).

**Table 3:** Testis volume and weight, and sperm count and motility in different groups of male Wistar rats

**Sperm motility (%)**	**Sperm count/ml (×10** ^4^ **)**	** Testis weight (g)**	**Volume of testis (cm** ^3^ **)**	**Groups / Mean±SEM**
72.05±2.59	1007±60.66	1.57±0.107	1.98±0.046	Control
75.30±1.25	950±35.36	1.40±0.050	2±0.066	Sham
20.96±1.86 ***###	507±39.91 ***###	0.68±0.023 ***###	1.29±0.052 ***###	DM
21.85±3.45 ***###	637±47.64 ***##	0.80±0.032 ***###	1.69±0.068 *#$$	DM+Ext. (50 mg/kg)
89.48±4.05 **#$$$+++	952±31.79 $$$++	1.19±0.159 $$	1.89±0.037 $$$	DM+Ext. (100 mg/kg)
94.53±1.82 **##$$$+++	876±38.59 $$$+	1.26±0.120 $$+	1.91±0.092 $$$	DM+Ext. (200 mg/kg)

*indicates a significant difference compared to the control group. # indicates a significant difference compared to the sham group. $ indicates a significant difference compared to the diabetic (DM) group. + indicates a significant difference compared to the DM + extract receiving (EXT; 50 mg/kg) group. (* p<0.05, ** p<0.01 and *** p<0.001) and (# p<0.05, ## p<0.01 and ### p<0.001) and ($$ p<0.01 and $$$ p<0.001) and (+ p<0.05 and +++ p<0.001).

**Table 4:** Serum levels of NO, and MDA, as well as SOD and CAT activity in different groups of male Wistar rats

**CAT (U/ml)**	**SOD (U/ml)**	**MDA (μmol)**	**NO (μmol/L)**	**Groups / Mean±SEM**
28.58±1.59	29.73±0.48	6.18±0.85	60.50±2.38	Control
25.72±1.08	28.50±0.57	8.55±0.87	63.22±0.77	Sham
13.21±0.50 ***	18.67±0.36 ***###	27.43±1.87 ***###	30.60±3.80 ***###	DM
16.95±1.14 ***	22.75±0.36 ***###$$$	20.97±0.83 ***###$$	38.98±4.78 ***###	DM+Ext. (50 mg/kg)
22.19±0.96 **###$$$++	23.22±0.62 ***###$$$	18.39±1.21 ***##$$$	34.60±2.74 ***###	DM+Ext. (100 mg/kg)
23.44±0.91 *###$$$+++	26.55±0.24 ***$$$+++**†††**	13.04±1.37 *$$$++**†**	54.60±1.93 $$$++**†††**	DM+Ext. (200 mg/kg)

*indicates a significant difference compared to the control group. # indicates a significant difference compared to the sham group. $ indicates a significant difference compared to the diabetic (DM) group. + indicates a significant difference compared to the DM + extract receiving (EXT; 50 mg/kg) group. **† **indicates a significant difference compared to the DM + Ext (100 mg/kg) group. (* p<0.05, ** p<0.01 and *** p<0.001) and (## p<0.01 and ### p<0.001) and ($$$ p<0.001) and (++ p<0.01 and +++ p<0.001) and (**†** p<0.01 and **†††** p<0.001).

## Discussion

It was reported that (Shiravi and Sayyad Zomorrodi, 2015 ▶; Zia-Ul-Haq et al., 2014 ▶) (Monforte et al., 2018 ▶) that raspberry fruit extract has antioxidant effects *in vivo* and *in vitro*. To determine the anti-diabetic effects of raspberry fruit extract (*Rubus fruticosus* L.) on pituitary-gonadal axis and testicular histopathology in STZ-induced diabetic male rats, we measured antioxidant capability of the raspberry fruit extract and evaluated the improving effects of the extract on the male reproductive system in diabetic rats. Our findings showed that the raspberry fruit extract can interestingly reverse the destructive alterations in the male reproductive system caused by diabetes in animals.

**The effects of diabetes on pituitary-gonadal axis**

In our experiment, chemically-induced diabetes in rats resulted in enhanced serum levels of LH and FSH but decreased serum levels of testosterone, and damaged testicular tissue and sperm quality and quantity. In line with our finding, previous studies have shown that diabetes leads to morphological damages to testicular tissue, and thus, alters serum level of gonadotropins (Sönmez et al., 2015 ▶). Higher levels of LH and FSH than the normal range are indicative of some pathologic processes (Hussein and Al-Qaisi, 2012 ▶). Previous research results revealed that diabetes mellitus can lead to a lower level of testosterone, reproductive glands atrophy, reduced sperm count, decreased sperm motility and reduced spermatogenesis in testicular tissue (Tafakkor et al., 2017 ▶). It was suggested that diabetes oxidative stress reduces the activity of the Leydig cells and leads to decreased synthesis and secretion of testosterone (Fernandez-Miro et al., 2016 ▶). Diabetes and its associated oxidative stress, by increasing the level of free radicals, and affecting LH and FSH receptors, reduce serum testosterone levels (Tirabassi et al., 2016 ▶) and with a negative feedback effect to adjust the serum testosterone level, the secretion of LH and FSH from the pituitary gland increases (Guyton and Hall, 2020 ▶).

**The improving effects of raspberry fruit extract on pituitary-gonadal axis**

Regarding our findings, raspberry fruit extract contains a considerable proportion of flavonoids which contribute to its improving effects on pituitary-gonadal system in diabetic animals. A large body of experimental studies revealed the improving effects of flavonoids on serum levels of gonadotropins and testosterone as well as testicular tissue and function (Ahangarpour et al., 2015 ▶). Aromatic compounds found in raspberries can increase norepinephrine (Morimoto et al., 2005 ▶). Norepinephrine, in turn, induces the release of GnRH from the hypothalamus and ultimately, FSH and LH secretion from the anterior pituitary gland by increasing NO synthesis (Selvage and Johnston, 2004 ▶; Całka, 2006 ▶). Gonadotropins increase testosterone secretion from Leydig cells. Testosterone with negative feedback on hypothalamus, reduces GnRH secretion which will eventually lead to a reduction in secretion of LH and FSH from the pituitary (Guyton and Hall, 2020 ▶), the story that is justifiable regarding our findings about gonadotropins, testosterone and NO in the diabetic group and diabetic animals treated with raspberry fruit extract.

**The improving effects of raspberry fruit extract on sperm count and motility**

Free radicals are produced through chemical reactions and oxidative stress occurring inside the cell (Khaki et al., 2014 ▶). The membrane of the spermatozoon has considerable amount of unsaturated fatty acids. These fatty acids are very sensitive to free radicals (Del Barco-Trillo and Roldan, 2014 ▶). Diabetes may result in lipid peroxidation, which plays a role in sperm motility reduction (La Vignera et al., 2012 ▶; Sanocka and Kurpisz, 2004 ▶). It has been reported that raspberry fruit, as a strong antioxidant, can reduce oxidative stress by removing free radicals (Giribabu et al., 2014 ▶). Although raspberry extract contains phenolic compounds (Zia-Ul-Haq et al., 2014 ▶), it may protect the testicular cells including Leydig cells from free radical effects (Nejati and Khaneshi, 2014 ▶). However, further investigations are required to show the mechanism by which raspberry fruit extract protects the Leydig cells from free radicals’ destructive effects.

**The effects of diabetes on NO, MDA, SOD and CAT**

The results of the present study revealed that serum NO level and SOD and CAT activity significantly decrease and serum MDA level increases in diabetic animals. Previous studies reported that diabetes can interfere with the secretion and synthesis of NO through vascular endothelial cells (Aghamohammadi et al., 2017 ▶). Decreases in NO level were reported in STZ-induced diabetic animals (Malekifard et al., 2016 ▶). The decrease of NO in diabetes may be brought about by an increase in angiotensin converting enzyme activity in serum and aorta of the diabetic animals (Sharifi et al., 2004 ▶), leading to increased angiotensin II production, followed by an increase in systolic blood pressure (Brands and Fitzgerald, 2002 ▶), resulting in a significant reduction in serum NO level (Heidari et al., 2006 ▶). Past studies showed that reducing the activity of antioxidant enzymes increases plasma concentrations of MDA (Hajinezhad et al., 2015 ▶). Diabetes induction with STZ was reported to decrease SOD and CAT activity (Khaki et al., 2014 ▶). Indeed, MDA, as a malignant product of lipid peroxidation, causes damage to testicular tissue (Salimnejad et al., 2014 ▶). Reduced SOD also results in apoptotic testicular death (Zhao et al., 2013 ▶), which would eventually lead to degeneration of testicular tissue and lower testosterone secretion, which in turn increase the secretion of gonadotropin hormones to compensate for this deficiency (Guyton and Hall, 2020 ▶).

**The improving effects of raspberry fruit extract on NO, MDA, SOD and CAT**

In our study, treatment of diabetic animals with raspberry fruit extract could reverse the alterations in serum level of NO and MDA, and SOD and CAT activity caused by chemical diabetes induced in animals. Chemical analysis of the raspberry fruit extract showed that the extract is rich in phenolic compounds. These compounds were reported to increase the secretion of testosterone from Leydig cells (Nejati and Khaneshi, 2014 ▶), leading to increased serum NO levels (Ahmadi and Solgi, 2004 ▶). However, the reports concerning effects of raspberry fruit extract on NO system are far limited. Therefore, further investigations on the impact of raspberry fruit extract on other cells than Leydig cells are required to reveal the pathways by which, the extract induces NO secretion. It seemed that flavonoid content and phenolic compounds in raspberry fruit extract played a significant role in reversing the pathological alterations observed in diabetic rats. Long-term administration of flavonoid-rich extracts in diabetic rats was reported to reduce serum MDA level (Hajinezhad and Mousavi, 2014 ▶) and increase levels of endogenous antioxidant enzymes including SOD and CAT (Giribabu et al., 2014 ▶). To the best of our knowledge, there is no previous study reporting the effects of raspberry fruit extract on MDA, SOD and CAT activity in diabetic animal models.

In conclusion, the raspberry fruit phenolic compounds- and flavonoid-rich extract has protective effects on pituitary-gonadal axis in diabetic animals, reversing the destructive alterations in serum level of NO and MDA and SOD and CAT activity as well as testicular tissue caused by chemically diabetes induction in animals.

Our findings revealed that the fresh fruit extract of the raspberry (*Rubus fruticosus* L.) can reverse the destructive alterations caused by diabetes in the male pituitary-gonadal axis and testicular tissue. This reversion is partially due to improving effects of the extract on NO system, and SOD and CAT activity. Although this study could not reveal the mechanisms behind the effects of the fruit extract of the raspberry on testicular tissue at cell and molecular level, the results are probably applicable in treatment of complications of diabetes occurring in the reproductive system. Further *in vitro* and *in vivo* as well as clinical trial research are needed to demonstrate antidiabetic effects of the fruit extract of the raspberry on the human reproductive system.
